# Research on the impact of digitalization on green development: An empirical analysis from the low-carbon strategy perspective

**DOI:** 10.1371/journal.pone.0300288

**Published:** 2024-03-08

**Authors:** Jiehui Zhang

**Affiliations:** Department of Financial Management, School of Economics and Management, Suqian University, Suqian City, Jiangsu Province, China; Qingdao University, CHINA

## Abstract

With the collision between the green and low-carbon economy and the accelerating digital economy, how to realize the effect of "1+1>2" has gradually become an important topic for contributing to the high-quality development of regions and enterprises. Entrepreneurship in the digital age continues to exhibit new characteristics, and its impact on green development is also more closely related. This article focuses on the context of the low-carbon strategy, incorporating the digitalization level, entrepreneurship, and green development into the same framework. It then takes 2011–2021 Chinese provincial panel data and enterprise panel data as samples to conduct research. The results indicate the following: (1) The digitalization level has a significant positive promoting effect on the green development of regions and enterprises, and blockchain technology has the strongest promoting effect on the green development of enterprises. (2) The digitalization level drives the green development of regions and enterprises through three channels: entrepreneurs’ innovative spirit, entrepreneurs’ entrepreneurial spirit and entrepreneurs’ contract spirit. Entrepreneurship is the intermediary bridge for the digitalization level to promote green development. (3) Environmental regulations partially serve as "accelerators" of the impact of green development. The findings of this article will provide empirical support for evaluating the impact of digitalization on green development and offer useful insights for better stimulating and cultivating entrepreneurship in the new era to empower comprehensive green development.

## Introduction

Climate change is a common challenge facing humanity today. With the increasingly prominent issue of global climate change, it is imperative for humanity to accelerate carbon reduction. In the Report of the 20th National Congress of the Communist Party of China, President Xi Jinping proposed "promoting green development and promoting harmonious coexistence between humans and nature", which is a major strategic deployment based on building a socialist modern power in all respects and achieving the second centenary goal. It also marks China’s gradual entry into a new stage of comprehensive green development. In August 2022, the Ministry of Science and Technology, the National Development and Reform Commission and nine other departments jointly issued the Implementation Plan for Science and Technology to Support Carbon Peaking and Carbon Neutrality (2022–2030). This policy document proposed scientific and technological innovation actions and safeguard measures to support the realization of the carbon peak, and it established technology research and development (R&D) reserves for achieving carbon neutrality by 2060. With the deep integration and application innovation of digital technology in the fields of resources, energy and the environment, the role of the digital economy in achieving the goal of carbon neutrality is receiving increasing attention [1]. Capturing the new opportunities offered by digital technology development and constantly promoting regional and enterprise green development with digital technology as support have become a hot topic of widespread concern.

Entrepreneurship in the digital age continues to exhibit new characteristics and a new essence. For a long time, entrepreneurship, which values integrity, a sense of responsibility, and the pursuit of sustainable development models, has played an important role in China’s economic construction [2]. In the era of the digital economy, Internet thinking characterized by innovation, equality, and connectivity has fundamentally converged with entrepreneurship [3]. The commercial civilization system of the new digital trust mechanism has also become the soil for cultivating entrepreneurial contract spirit. In the context of the low-carbon strategy, entrepreneurs will face new opportunities for green transformation, such as low-carbon technologies, products, and models, and their impact on green development will also be more closely related. The green development of cities and enterprises is facing a new situation, and there should be a new way to resolve the contradiction between the demand for a green and low-carbon transformation and the increase in energy demand to realize economic growth. Therefore, exploring whether entrepreneurship can become a bridge from the digitalization level to green development is an important topic for guiding all parties to shift carbon reduction from concept to practice.

To further explore the process mechanism of how the digitalization level of macro and micro subjects promotes green development and open the "black box" of the relationship between the two, this study incorporates digitalization level, entrepreneurship and green development into the same analytical framework, then takes 2011–2021 Chinese provincial panel data and enterprise panel data as samples to explore the relationship between the digitalization level and green development as well as the mediating role of entrepreneurship from the macro and micro perspectives. Furthermore, this study also incorporates environmental regulations as a moderating variable to test the contingency impact of this path. The innovation and potential contributions of this article are as follows. First, this study focuses on the important role of entrepreneurship in promoting green development though digitalization, deepening the theoretical exploration of green development from the perspective of endogenous factors, and to some extent compensating for the shortcomings of existing literature. Second, with the integration and development of the digital economy and the ecological economy, the connotations of the digitalization level and green development have become more abundant. This study measures and analyzes them from both macro and micro levels, expanding and enriching their theoretical connotations, and also providing corresponding evidence for a more comprehensive and objective understanding of the relationship between the two. Finally, from a practical perspective, this article provides empirical support for better stimulating and cultivating entrepreneurship in this new era to empower comprehensive green development, guiding all parties to shift carbon reduction and green development from concept to practice.

## Literature review

### Research on green development

Pearce first proposed the concept of the “green economy” in 1989 [4]; he defined it as a "sustainable economy" established from the perspective of society and its ecological conditions. Subsequently, the concept of sustainable development became a hot topic of research among scholars. With the global economic crisis, climate damage, biodiversity, and pollution issues are becoming increasingly severe, and the concept of environmental, social, and governance (ESG) responsibility has emerged in research both in China and elsewhere. In September 2020, China clearly proposed the goals of "carbon peaking" by 2030 and "carbon neutrality" by 2060, and Chinese research on green development has also been expanding from the perspectives of its definition, measurement dimensions, influencing factors, driving mechanisms, etc. Most scholars discuss the impact mechanism and improvement path of green development at the macro level of the government, cities, the environment, technology and other aspects in combination with theories and models from industrial economics [5–8]. Research shows that the level of macro green development is closely related to the industrial structure, the level of technological innovation, institutional arrangements, green finance development and other factors. Some scholars have also studied the green development strategy and behavior of enterprises at the micro level, finding that enterprises’ implementation of green development has a significant promoting effect on their economic and social benefits [9–12].

### Research on the digitalization level

Don Tapscott first proposed the concept of the “digital economy” in 1995, and Negroponte [13] also began using the term "digitalization" in his work in 1995. Since then, research on the impact of the Internet on the economy and society has continued to emerge. In 2015 and 2017, the "Internet plus" and "digital economy" concepts were first proposed in China’s Report on the Work of the Government, and then, research on the digital economy, digital transformation and other issues began to attract the attention of Chinese scholars. From the literature, some scholars have provided theoretical support and evidence support for the impact of digitalization on China’s economic growth, industrial upgrading, regional employment optimization, and innovation issues from the macro perspective combined with measurement research on the digitalization level [14–17]. More scholars have focused on the micro impact of the digitalization level from the perspective of enterprises, demonstrating through empirical testing the impact of the digitalization level on enterprise economic performance and innovation as well as the mechanism of the impact [18–21]. At the same time, some scholars have paid attention to the impact of digitalization on noneconomic performance, such as corporate social responsibility, the governance level, carbon emission reduction, and carbon performance [22,23]. This literature review shows that the level of digitalization not only brings new growth space to the regional macroeconomy and industrial development but also improves the economic and noneconomic benefits of enterprises to a certain extent.

### Research on the impact of digitalization level on green development

From an industry perspective, research on the impact of digitalization on green development mainly focuses on manufacturing and agriculture. Xiao et al. [15] used provincial balanced panel data as samples to measure the green total factor productivity of the manufacturing industry, and they empirically tested whether the regional digitalization level enables the green transformation and development of the manufacturing industry through green technology innovation. Fan et al. [24] focused on the spatiotemporal characteristics of China’s interprovincial agricultural green development level and found an inverted U-shaped relationship between the digitalization level and agricultural green development. Regarding research methods, most scholars use quantitative empirical research methods, while others use case studies. For example, Cao et al. [25] combined resource allocation theory and adopted a vertical single-case approach in their research process to find that the digital behavior of enterprises can activate digital features and enhance different key capabilities of enterprises, thereby driving the green transformation and development of manufacturing enterprises.

In summary, compared to the booming digital economy, there is relatively little research on the impact of digitalization level on green development, and the literature on this issue mainly focuses on external influencing factors and their economic value impact. Few studies examine the entrepreneurship that can stimulate the vitality of the market and enterprises. The core of current theoretical and practical research is the external driving force of green development, however relying solely on it to promote green development cannot form a sustainable and stable development momentum. Therefore, it is essential to explore green development in the context of digitalization level and entrepreneurship and to reveal the implementation paths from macro and micro perspectives, which will provide theoretical support and empirical evidence for promoting comprehensive green development.

## Theoretical analysis and research hypotheses

### The digitalization level and green development

Digital technology is an important carrier for the development of the digital economy. In the face of an uncertain economic environment and energy conservation and emission reduction paths, the integration and application of technologies such as big data and cloud computing will effectively promote the determination and effective implementation of green development strategies for the economy and enterprises. First, digital technology can effectively realize precise measurement and long-term predictions of carbon emissions, guiding cities and enterprises to prioritize green development strategies with low-carbon goals. At the same time, big data can be used to obtain and accurately identify ecological environment information in real time, enabling cities and enterprises to assess the capacity and situation of the environment [26], and big data can also help them fully tap into the value of carbon sink assets and other assets [27]. The precise planning of carbon emission reduction and carbon neutrality combined with digital twin technology can also eliminate the adverse selection and moral hazard caused by asymmetric information and force subjects to make a strategic plan for green development in advance.

Second, digital technologies such as the Internet of Things and artificial intelligence (AI) can guide green energy consumption through innovative energy formats and promote the green development of the economy and enterprises from the supply and demand sides. In the supply chain, a new round of energy technology innovation effectively drives green technology innovation in enterprises, empowering the green economic development of the entire industry chain through forms such as clean technology transformation, management model innovation and the transformation of energy structure [18,28,29]. Such innovation not only innovates existing production models but also builds a clean, low-carbon, safe and efficient energy system. At the same time, cities and enterprises can use technologies such as the Internet of Things to improve their energy collection efficiency and online connectivity, achieve intensive, data-driven, and refined energy supply links, further promote efficient energy scheduling and utilization, and reduce their energy consumption costs. In the consumption sector, digital technologies such as AI will replace traditional energy consumption concepts, promote new energy consumption methods, lead to consumption changes in various industries, and reduce energy consumption and intensity [1].

Third, blockchain technology will help to achieve efficient operation of the energy market and incentives for low-carbon behavior, thereby mitigating the externalities for the environment. Blockchain technology can promote the innovation of the distributed energy market by facilitating resource allocation, secure and reliable trading and the efficient settlement of the energy trading market [1]. Furthermore, the unique incentive mechanism of blockchain is similar to a work point system, which can solve the environmental externality problem and stimulate green development behavior to a certain extent by giving rights-based incentives, increasing revenue-based incentives and improving reputation-based incentives [30].

Based on the analysis above, this article proposes the following research hypothesis:

**H1:** *The digitalization level has a significant positive promoting effect on the green development of regions and enterprises*.

### The mediating role of entrepreneurship

Audretsch et al. [31] first proposed the Entrepreneurship Capital Theory, which is considered a new development of Endogenous Growth Theory. According to this theory, Entrepreneurship is regarded as a key link connecting knowledge and sustainable growth, and has also become an important endogenous driving force for promoting development. Both the "father of modern management" Peter Drucker and the "ancestor of innovation theory" Joseph Schumpeter believed that innovation was the essence of entrepreneurship. From a macro perspective, the improvement in the digital development level has promoted the flow of talent, technology, and knowledge within a region [32], providing a low-cost and efficient foundation for entrepreneurs to carry out innovative activities. With the continuous gathering of innovative spirit among entrepreneurs in a region, entrepreneurs can pay more attention to new business formats in the energy market and the opportunity space in the green consumption market, thereby innovating the effective allocation of resources and reducing the transaction costs of innovative entities in the region. This can promote the transformation of consumption within the industry and continuously promote regional green development. From a micro perspective, the digital construction and application of enterprises stimulate the innovative spirit of entrepreneurs by creating an open innovation ecosystem, thereby promoting green technology innovation, organizational management innovation, and other behaviors to promote the green development level of enterprises. In the new organizational form of the open innovation ecosystem, the spillover effect of innovative knowledge and thinking in enterprises is constantly updated and iterated under the strong penetration of digital technology [33]. Systems such as big data and intelligent decision-making also further improve the success rate of innovation. With the improvement in the innovation atmosphere of enterprises, enterprises can quickly capture new opportunities for their green transformation, such as low-carbon technologies, products, and models. Subsequently, the integration of digital technology and other elements such as energy is further deepened, promoting the restructuring of enterprise production factors, introducing new systems into organizational innovation, and realizing the level of green development of enterprises by driving green technology innovation [19].

Another important characteristic of entrepreneurship is the tendency to identify business opportunities and create new firms, which are concentrated in the entrepreneurial spirit. From a macro perspective, digital development has stimulated market vitality and consumer demand. It cultivates more entrepreneurial opportunities by influencing market size, knowledge spillovers, and factor combinations, and it enriches entrepreneurial resources by accelerating information exchange and spreading ideas. The sharing of the labor force between industries, the proximity of upstream and downstream markets, and the knowledge spillovers between industries have led to increased entrepreneurial opportunities, improved efficiency in market resource allocation, and increased cultural capital, all of which positively affect local entrepreneurs’ entrepreneurial spirit [34]. With the continuous gathering of active entrepreneurial enterprises in a region, open competition is becoming increasingly fierce, and the degree of marketization is also constantly improving, forming a sufficient market competition mechanism in the industry [35]. These changes force regional entities to pay attention to "green" sustainable development concepts such as the environment and society in the development process, establish green development strategies in advance, and continuously promote the level of green development. From a micro perspective, with the continuous optimization of the entrepreneurial environment, the "imitation-oriented" and "adventure-oriented" pioneering spirit of entrepreneurs is further stimulated. In the early stages of entrepreneurial activities, entrepreneurs can quickly capture entrepreneurial opportunities through market gaps, reshape personal social networks, and stimulate entrepreneurial activities through new financing methods such as digital finance [36]. In the process of carrying out entrepreneurial activities, entrepreneurs can use the sharing of digital economy technology to more accurately identify potential risks, reduce internal and external information asymmetry, and improve the success rate. In the later stages of entrepreneurial activities, the promotion of digital economy platforms can effectively reduce financing constraints [37] and to some extent solve the problem of restrictions on subsequent entrepreneurial funds. Therefore, the entrepreneurial activities of entrepreneurs can continuously promote the transformation of green technology achievements and solve the imbalance between the supply and demand of green consumption in enterprises, thus forming an important driving force for promoting the green development of enterprises in low-carbon transformation and shared development.

Entrepreneurship in the digital age constantly showcases new characteristics and a new essence [3]. From a macro perspective, credit is the lubricant for the construction and operation of a market economy. The credit evaluation mechanism based on the digital economy has become an important part of building a social credit system [38]. As the soul of the credit system, the contract spirit that focuses on the relationship between enterprises and society has gradually become an important characteristic of entrepreneurs in the new era. The concentration of contract spirit within a region reduces the uncertainty and insecurity factors in the contract execution process and reduces the risks and costs brought by information asymmetry in contract performance. The entrepreneurial contract spirit not only plays a positive role in promoting an improvement in the regional innovation level and achievement transformation [39] but also enhances the social recognition and product satisfaction of enterprises. It helps the industry create a harmonious development space, ecological environment, and social foundation [40], becoming the cornerstone for promoting regional green development reform. From a micro perspective, digital technologies such as blockchain help enterprises quickly establish their own credit databases and establish stable credit cooperation relationships from the external perspective. Subsequently, from the internal perspective, digital technologies help enterprises create a healthy and harmonious organizational system, create a fair and honest work atmosphere, and provide a good foundation for the cultivation of entrepreneurs’ contract spirit. Under a smooth two-way feedback and communication mechanism, entrepreneurs pay more attention to corporate products and commercial civilization images such as "green energy" and "environmental protection and low-carbon", they pay attention to contracts between enterprises and employees, and they actively fulfill the promises made by enterprises to employees [3]. The accumulation of entrepreneurial contract spirit in the long run will drive entrepreneurs to adhere to the ecological protection, green and low-carbon concepts and to participate in external collaboration [41], leading enterprises to build a green development business reputation.

Based on the analysis above, this article proposes the following research hypotheses:

**H2:** *The digitalization level can further stimulate entrepreneurship*, *thereby promoting the green development of regions and enterprises*.
**H2a:** *The digitalization level can further stimulate entrepreneurs’ innovative spirit*, *thereby promoting the green development of regions and enterprises*.
**H2b:** *The digitalization level can further stimulate entrepreneurs’ entrepreneurial spirit*, *thereby promoting the green development of regions and enterprises*.
**H2c:** *The digitalization level can further stimulate entrepreneurs’ contract spirit*, *thereby promoting the green development of regions and enterprises*.

### The moderating role of environmental regulations

As an important lever in ecological civilization construction, government departments formulate and implement a series of rules and regulations for environmental governance and protection guided by optimizing the ecological environment and achieving sustainable development. The promulgation and implementation of these regulations have brought pressure and constraints to the development of cities and enterprises while also creating market opportunities for green development. The government’s implementation of effective environmental policies and regular environmental performance assessments can strengthen the innovative and pioneering spirit of entrepreneurs in identifying, developing, and utilizing specific social and environmental opportunities, making green industries a new market opportunity overflow point. Furthermore, to meet the requirements of environmental regulations, some heavily polluted regions and polluting enterprises will mobilize their own resources to improve their environmental performance by strengthening the spirit of entrepreneurial responsibility contracts, thereby achieving green upgrading and development [42]. When enterprises face high environmental regulatory pressures, entrepreneurship will sensitively perceive and search for more external, high-quality green knowledge and information. By optimizing the allocation of organizational green resources and improving the process of green technology innovation, enterprises will ultimately respond to changes and demands in the external environment with a responsible attitude, thereby promoting green development transformation.

Based on the analysis above, this article proposes the following research hypothesis:

**H3**: *Environmental regulations play a significant positive moderating role in the impact of entrepreneurship on green development*.

## Research design

### Model specification

Based on the analysis above, in order to verify the impact of digitalization level on green development, and considering the heterogeneity of research subjects in terms of individual and time, this article refers to existing relevant literature [3,20] and constructs the following benchmark model (1). Then this paper draws on the mediating effect test procedure proposed by Wen and Ye [43] to further clarify the mechanism of the impact of the digitalization level on green development and to test whether the digitalization level will affect green development through entrepreneurship. On the basis of Model (1), this article constructs Model (2) to test the impact of the digitalization level on entrepreneurship and Model (3) to test the impact of the digitalization level and entrepreneurship on green development. Considering that external environmental regulations may have a moderating effect on the impact of entrepreneurship and green development, this article establishes the Model (4) by constructing an interaction term between entrepreneurship and environmental regulations.


$$GreenIndex_{i,t}=\alpha_{0}+\alpha_{1}Szfix_{i,t}+\alpha_{2}Controls_{i,t}+\mu_{i}+\delta_{t}+\varepsilon_{i,t}$$ (1)

$$ESP_{i,t}=\beta_{0}+\beta_{1}Szfix_{i,t}+\beta_{2}Controls_{i,t}+\mu_{i}+\delta_{t}+\varepsilon_{i,t}$$ (2)

$$GreenIndex_{i,t}=\theta_{0}+\theta_{1}Szfix_{i,t}+\theta_{2}ESP_{i,t}+\theta_{3}Controls_{i,t}+\mu_{i}+\delta_{t}+\varepsilon_{i,t}$$ (3)

$$GreenIndex_{i,t}=\gamma_{0}+\gamma_{1}ESP_{i,t}+\gamma_{2}ER_{i,t}+\gamma_{3}ESP_{i,t}\times ER_{i,t}+\gamma_{4}Controls_{i,t}+\mu_{i}+\delta_{t}+\varepsilon_{i,t}$$ (4)

When conducting macrolevel tests, GreenIndex_i,t_ represents the green development level of province i during period t; Szfix_i,t_ represents the digitalization level of province i during period t; Controls_i,t_ is a series of provincial-level control variables; μ_i_ represents the time-invariant individual fixed effect of province i; δ_t_ is the year fixed effect; and ε_i,t_ represents the random error term. According to model (1), α_1_ is the coefficient of Szfix_i,t_, which reflects the average impact of the digitalization level on regional green development. ESP_i,t_ represents the entrepreneurship of province i during period t. In this article, it is specifically divided into entrepreneurs’ innovative spirit, entrepreneurs’ entrepreneurial spirit, and entrepreneurs’ contract spirit. ER_i,t_ represents the environmental regulation intensity of province i during period t.

When conducting microlevel tests, GreenIndex_i,t_ represents the green development level of enterprise i during period t; Szfix_i,t_ is the digitalization level of enterprise i during period t; Controls_i,t_ is a series of control variables at the company level; μ_i_ represents the time-invariant individual fixed effect of enterprise i; δ_t_ is the year fixed effect of the year; and ε_i,t_ represents the random error term. α_1_ reflects the average impact of the digitalization level of enterprises on the green development of enterprises. ESP_i,t_ represents the corresponding spirit of enterprise i during period t.

### Setting and explanation of the main variables

#### Dependent variable: Green development

Some studies consider green total factor productivity or green economic efficiency as a proxy indicator for green development [15,44], but their evaluation dimension is relatively singular and cannot fully reflect the interaction between the economy, nature, and social dynamics. Therefore, we refer to the research of Hu and Zhou [45] and Wang et al. [46], focusing on green growth, green welfare, green wealth, and green governance. From these four dimensions, 19 indicators are selected to evaluate the level of green development in a region. The specific indicators are shown in Table 1. Among them, we take the reciprocal of the negative indicator and normalize the dimensionless data. Regarding the methodology, we use the panel entropy method to quantitatively measure the green development of 30 regions (except Tibet) in mainland China from 2011 to 2021 and obtain the green development level GreenIndex1. At the micro enterprise level, we refer to the research of Lin and Li [10] and Qiu and Yin [47] and select ESG performance as the indicator to measure the green development of enterprises. Based on data from the Huazheng ESG rating system, we assign a score of 1–9. Considering the impact of different industry characteristics on enterprise ESG scores, this article standardizes enterprise ESG scores by industry and obtains the GreenIndex2 index at the micro level.

**Table 1:** Macro indicator system for green development and the digitalization level.

Indexes	Dimensions	Index Definition and Unit	Index Attribute
GreenIndex	Green Growth	Per capita GDP (ten thousand yuan)	positive (+)
		Per capita general budget revenue of local finance (ten thousand yuan)	positive (+)
		Proportion of the value added of the tertiary industry (%)	positive (+)
		Electricity consumption per unit of GDP (KWh/ten thousand yuan)	negative (-)
		SO_2_ emissions per unit of GDP (ton)	negative (-)
		Wastewater discharge per unit of GDP (ton)	negative (-)
	Green Welfare	Per capita retail sales of social consumer goods (yuan)	positive (+)
		Per capita disposable income (yuan)	positive (+)
		Scientific research funds for industrial enterprises above a designated size (ten thousand yuan)	positive (+)
		Number of college students per 10,000 people	positive (+)
		Bus ownership per 10,000 people	positive (+)
	Green Wealth	Per capita water resources (cubic meter)	positive (+)
		Per capita forest area (hectare)	positive (+)
		Per capita natural reserve area (hectare)	positive (+)
		Green coverage rate of built-up areas (%)	positive (+)
		Per capita park green area (cubic meter)	positive (+)
	Green Governance	Amount of completed investment in industrial pollution control (ten thousand yuan)	positive (+)
		Urban sewage treatment rate (%)	positive (+)
		Harmless treatment rate of household waste (%)	positive (+)
Szfix	Economic Infrastructure	Unit length of long-distance optical cable lines (kilometers/square kilometers)	positive (+)
		Internet broadband access ports for every 100 people (ten thousand/one hundred persons)	positive (+)
		Mobile phone exchange capacity per household (%)	positive (+)
	Development Scale	Per capita express business volume	positive (+)
		Per capita total telecommunications business (ten thousand yuan)	positive (+)
	Application Level	Proportion of mobile Internet users (%)	positive (+)
		Internet penetration rate (%)	positive (+)
		Proportion of personnel in the information transmission, software, and information technology service industries (%)	positive (+)

#### Independent variable: The digitalization level

This article refers to the research of Zhao et al. [48], Huang et al. [49], and Yu and Wang [3] and constructs an indicator system from three dimensions: digital economy infrastructure, the development scale, and the application level. This indicator is used to comprehensively measure the level of regional digitalization. The specific indicators are shown in Table 1. Subsequently, regarding the methodology, we use principal component analysis for dimensionality reduction to obtain the regional digitalization level index Szfix1. At the micro enterprise level, to objectively and reasonably measure the technology and digital strategic orientation of enterprises, this article refers to the approach of Wu et al. [50]. We confirm keyword roots at the application and technological levels, eliminate expressions of negative bestowal before keywords, and then use Python software to identify and count word roots. Finally, we take the logarithmic form of "digitalization" in the annual reports of listed companies and obtain the proxy indicator Szfix2 for the digitalization level of enterprises.

#### Mediating variable: Entrepreneurship

Based on the theoretical analysis and research assumptions above, this article argues that the impact of entrepreneurship on the digitalization level and green development is mainly manifested in entrepreneurs’ innovative spirit, entrepreneurs’ entrepreneurial spirit, and entrepreneurs’ contract spirit. The following text analyzes and measures these three dimensions from the macro and micro perspectives.

1. Entrepreneurs’ innovative spirit: This article draws inspiration from the research of Li et al. [51], Wong et al. [52], and Yu and Wang [3] and measures the innovative spirit of provincial-level entrepreneurs by dividing the number of granted invention patents, utility model patents, and design patents by the gross domestic product, denoted as ESP1. At the micro level, we draw inspiration from the research method of Cheng [53] and measure entrepreneurs’ innovative spirit at the enterprise level by the ratio of R&D investment to operating income in the current year, denoted as ESP4.
2. Entrepreneurs’ entrepreneurial spirit: At the macro level, the literature selects the enterprise ownership ratio, enterprise entry rate, and exit rate [54,55] or the number of new startups for measurement. This article draws on the research of Li et al. [51] and Yu and Wang [3]. We select the proportion of the number of employees in individual units and private enterprises to the total number of employees as the proxy indicator for measuring entrepreneurial spirit at the provincial level, denoted as ESP2. At the micro level, this article draws on the research of Li et al. [56] and selects the management shareholding ratio as the proxy indicator for entrepreneurial spirit, denoted as ESP5.
3. Entrepreneurs’ contract spirit: This article refers to the research of Liu et al. [57] and Yu and Wang [3], and we use the "China Urban Commercial Credit Environment Index" (CEI) to measure entrepreneurs’ contract spirit, denoted as ESP5. This index is the outcome of a comprehensive evaluation conducted by the Chinese Academy of Management Sciences, focusing on six dimensions: credit placement, the credit reporting system, government credit supervision, dishonest and irregular behavior, enterprise credit management, and integrity education. At the micro level of the enterprise, contract spirit can mainly be measured from the perspective of contracts with employees. Therefore, this article refers to the research of Yang [58] and measures entrepreneurs’ contract spirit using the wages and benefits paid to employees, denoted as ESP6.

#### Moderating variable: Environmental regulations

From the literature, there are three main methods for quantifying the intensity of environmental regulations at the macro level: cost-based, performance-based, and index-based methods. Considering the availability, objectivity, and completeness of data, this article refers to the research of Yang et al. [59] and Zhang and Cai [60], uses the proportion of regional industrial pollution control investment to industrial value added to measure the intensity of environmental regulation, and then performs decentralized processing. The variable is denoted as ER.

#### Control variables

Referring to the literature, at the macro level, this article selects the following control variables that may have an impact on green development: (1) the resource endowment (Res), measured as the proportion of mining industry employees to the total number of employees; (2) the financial development level (Fin), measured as the ratio of the year-end deposit and loan balances of financial institutions to GDP; (3) the infrastructure level (Tra), measured as the ratio of the sum of mileage of highways and operating railways in each region to the area of each province; and (4) government size (Gov), measured as the ratio of local government general budget expenditure to regional GDP. At the micro level, the following variables are controlled for: (1) enterprise size, which is measured by the natural logarithm of total assets at the end of the period; (2) enterprise growth, measured as the total asset growth rate; (3) enterprise age, measured as the natural logarithm of the years being listed; (4) the asset liability ratio (Lev), measured as the ratio of a company’s total liabilities to its total assets; and (5) property rights nature (State), where if the ultimate control of the enterprise lies with the state (i.e., a state-owned enterprise), this variable takes the value of 1 0 otherwise.

### Data sources and descriptive statistical analysis of the variables

This paper uses Chinese provincial panel data in the empirical analysis at the macro level. The sample includes 30 provinces in mainland China (excluding Hong Kong, Macao, Taiwan and Tibet), and the time span is 2011–2021. All kinds of data are mainly from the website of the National Bureau of Statistics, the China Statistical Yearbook and the China Environmental Statistical Yearbook over the years. When conducting empirical analysis at the micro level of enterprises, we select A-share listed companies from 2011 to 2021 as the research objects, with the main data coming from the China Stock Market and Accounting Research (CSMAR) database, CNINFO and the official website of the Shanghai China Securities Index. Regarding data processing, we use a combination of the annual report text analysis method and annual report financial data method. Then, based on the research purpose, we exclude some samples, including ST and *ST companies, enterprises in the financial industry, samples that were unable to continue operations during the period, and samples with severe data deficiencies. Finally, we obtain a total of 26,700 valid sample data points. After conducting bilateral 1% tail reduction on the continuous variables, this article uses Stata 16.0 econometric analysis software to process and report the data. The descriptive statistical analysis results are shown in Table 2, and the characteristics of each variable are relatively stable, providing a certain foundation for empirical analysis.

**Table 2:** Descriptive statistics of the main variables.

Variables		Mean	Standard Deviation	Maximum	Minimum	Number
Dependent Variables	GreenIndex1	0.158	0.067	0.466	0.065	330
	GreenIndex2	6.33	1.369	9	0.000	26700
Independent Variables	Szfix1	2.344	1.497	8.012	0.010	330
	Szfix2	1.449	1.423	6.301	0.000	26700
Mediating Variables	ESP1	1.819	1.312	7.013	0.158	330
	ESP2	0.412	0.216	1.277	0.120	330
	ESP3	71.904	4.781	90.630	65.921	330
	ESP4	4.915	7.373	424.930	0.000	26700
	ESP5	16.147	20.853	89.99	0.000	26700
	ESP6	10.096	1.303	16.565	0.000	26700
Moderating Variable	ER	0.003	0.004	0.031	0.00008	330
Control Variables (macro level)	Res	0.127	0.284	1.109	0.00008	330
	Fin	3.276	1.150	8.131	1.518	330
	Tra	0.924	0.523	2.188	0.065	330
	Gov	0.249	0.103	0.643	0.107	330
Control Variables (micro level)	Size	12.917	1.308	19.426	8.067	26700
	Growth	0.224	0.597	33.060	-0.961	26700
	Age	8.84	7.425	31	0	26700
	Lev	0.405	0.484	63.971	0.008	26700
	State	0.30	0.457	1	0	26700

## Empirical results and analysis

### Benchmark regression results

To verify the impact of digitalization on green development, this article uses Model (1) and relevant measurement data to conduct benchmark regression analysis on the research hypothesis. The results are shown in Table 3. The data in columns (1) and (2) correspond to the regression results before and after adding the control variables at the macro level, respectively. We find that the fitting degree of the models exceeds 75%, and the coefficient of the digitalization level is always positive and significant at the 1% level. From the correlation coefficient, it can be seen that for every 1% increase in the regional digitalization level, the green development level increases by 0.03%. Columns (3) and (4) are the regression results at the micro level of the enterprise. We find that the model fitting degree is significantly optimized after adding the control variables, and the coefficient of the digitalization level is constantly decreasing, indicating that the results have a certain robustness and the selection of the control variables has a certain representativeness. Specifically, the coefficient of the digitalization level is always positive and significant at the 1% level. When the digitalization level of an enterprise increases by 1%, its green development level increases by 0.04%. In summary, the level of digitalization has a significant promoting effect on green development, and H1 is confirmed.

**Table 3:** Benchmark regression results of the effect of the digitalization level on green development.

Variables	Macro level		Micro level	
	(1)	(2)	(3)	(4)
	GreenIndex1	GreenIndex1	GreenIndex2	GreenIndex2
Szfix1	0.0261***	0.0325***		
	(29.18)	(21.57)		
Szfix2			0.0436***	0.0405***
			(5.19)	(7.31)
Res		-0.0054*		
		(-1.74)		
Fin		-0.0128***		
		(-3.57)		
Tra		-0.0123		
		(-1.22)		
Gov		-0.0773*		
		(-1.76)		
Growth				-0.01728***
				(-12.95)
Age				0.0088***
				(6.81)
Lev				-0.1837***
				(-11.15)
State				0.3517***
				(17.27)
Constant	0.0971***	0.1553***	6.2698***	2.6340***
	(42.60)	(12.05)	(460.3)	(31.16)
Province/Firm fixed effect	Yes	Yes	Yes	Yes
Year fixed effect	Yes	Yes	Yes	Yes
R^2^	0.7401	0.7772	0.0012	0.3151
Observations	330	330	26700	26700

Notes. ***p < 0.01; **p < 0.05; *p < 0.1. The t value is in brackets.

### Robustness tests

#### Replacing the core dependent variable

This study first refers to the approach of Li et al. [61] and replaces the panel entropy method with principal component analysis to recalculate the level of regional green development. Then, we use the enterprise ESG performance data provided by Bloomberg Consulting to replace the Huazheng enterprise ESG performance data used in the benchmark model for regression. The regression results are shown in Table 4, showing that the models have a good fit. The digitalization level regression coefficient at the macro level is positive and significant at the 1% level. After replacing the measurement method for measuring the green development level of micro enterprises, the regression coefficient of the digitalization level remains positive and significant. In summary, the conclusion that the digitalization level has a significant promoting effect on green development has a certain robustness.

**Table 4:** Test results of replacing the dependent variable.

Variables	Macro level	Micro level
	GreenIndex1	GreenIndex2
Szfix1	0.9461***	
	(27.37)	
Szfix2		0.1785***
		(3.69)
Controls	Yes	Yes
Province/Firm fixed effect	Yes	Yes
Year fixed effect	Yes	Yes
R^2^	0.8816	0.4347
N	330	26700

Notes.

***p < 0.01; **p < 0.05; *p < 0.1. The t value is in brackets.

#### Replacing the core independent variables

This paper conducts another robustness test by replacing the core explanatory variables. At the macro level, we refer to the approach of Huang and Zhu [62] and normalize the digitalization level indicator data of 30 provinces. Then, we use the panel entropy method to obtain the digitalization level index Szfix1. At the micro enterprise level, referring to the approach of Wu et al. [50], we construct a digital horizontal dictionary from five dimensions: "artificial intelligence technology", "big data technology", "cloud computing technology", "blockchain technology", and "digital technology application". We use Python software for root recognition and counting and take the logarithmic form of the word frequency of the five dimensions. The regression results are presented in Table 5, showing that the digitalization level coefficient at the macro level is 0.2563 and is significant at the 1% level. At the micro level, all subindicators of the digitalization level promote the green development performance of enterprises at a significance level of 1%. Among them, the impact coefficient of blockchain technology on the green development level of enterprises is 0.1202, which is much greater than the impact coefficient of other digital technologies. The reason may be that blockchain technology is characterized by centralization, openness, distrust, and collective maintenance. When applied to the production and operation processes of enterprises, it can not only alleviate the financing constraints of enterprises and reduce transaction costs but also enhance the expected benefits of enterprises undertaking environmental protection and other social responsibilities through traceability mechanisms. Therefore, compared to other technologies, the promoting effect of blockchain technology on the green development of enterprises is more obvious. In summary, we believe that the conclusions obtained from the benchmark regression model in this article are relatively robust and reliable.

**Table 5:** Test results of replacing the explanatory variables.

Variables	Macro level		Micro level				
	GreenIndex1	GreenIndex2		GreenIndex2	GreenIndex2	GreenIndex2	GreenIndex2
Szfix1	0.2563***						
	(14.56)						
Szfix21		0.0390***					
		(3.84)					
Szfix22				0.1202***			
				(2.63)			
Szfix23					0.0607***		
					(7.82)		
Szfix24						0.0555***	
						(6.54)	
Szfix25							0.0464***
							(6.63)
Controls	Yes	Yes		Yes	Yes	Yes	Yes
Province/Firm fixed effect	Yes	Yes		Yes	Yes	Yes	Yes
Year fixed effect	Yes	Yes		Yes	Yes	Yes	Yes
R^2^	0.6660	0.1338		0.1334	0.1352	0.1346	0.1346
N	330	26700		26700	26700	26700	26700

Notes.

***p < 0.01; **p < 0.05; *p < 0.1. The t value is in brackets.

### Endogenous test

The empirical testing process in this article may have endogeneity problems caused by mutual causality, or there may be bias in the endogeneity estimation caused by omitted variables. Therefore, we use lagged independent variables to handle these endogeneity problems. Considering that the impact of the digital development level may have a certain lag in time, this article introduces the lag of the core explanatory variables from one to three periods into the model for regression. The empirical results are shown in Table 6. The estimated coefficients of the digitalization levels at both the macro and micro levels shown in columns (1)-(6) are positive and significant at the 1% level. Therefore, the core conclusion of hypothesis H1 in this article is relatively reliable.

**Table 6:** Endogeneity test results.

Variables	Macro level			Micro level		
	(1)	(2)	(3)	(4)	(5)	(6)
	Green1	Green1	Green1	Green2	Green2	Green2
L.Szfix1	0.0442***					
	(13.19)					
L2.Szfix1		0.0492***				
		(12.84)				
L3.Szfix1			0.0531***			
			(12.19)			
L.Szfix2				0.0352***		
				(6.83)		
L2.Szfix2					0.0228***	
					(3.99)	
L3.Szfix2						0.0188***
						(2.87)
Controls	Yes	Yes	Yes	Yes	Yes	Yes
Province/Firm fixed effect	Yes	Yes	Yes	Yes	Yes	Yes
Year fixed effect	Yes	Yes	Yes	Yes	Yes	Yes
R^2^	0.4934	0.4976	0.4979	0.1487	0.1859	0.1805
N	300	270	240	22029	18442	15343

Notes.

***p < 0.01; **p < 0.05; *p < 0.1. The t value is in brackets.

## Mechanism research

To further open the black box between the digitalization level and green development and to clarify the path and mechanism of entrepreneurship in the process of the impact of the digitalization level on green development, this paper uses models (2) and (3) to conduct research, and it uses the Sobel test method to conduct a robustness test.

### The mediating effect based on entrepreneurs’ innovative spirit

Benefiting from the continuous deepening of digital construction and application, the flow of talent, technology, and knowledge within regions and enterprises has accelerated. Entrepreneurs can pay more attention to new business formats in the energy market and the opportunity space in the green consumption market, which can promote consumption transformation within the industry and help enterprises effectively carry out green technology innovation and other activities. This makes innovation spirit an important driving force for promoting green development. According to the empirical results data in Table 7, an improvement in the digitalization level at the macro and micro levels can significantly stimulate the innovative spirit of entrepreneurs, thereby driving the continuous improvement in regional and enterprise green development. After Sobel testing, it is found that there is a positive transmission mechanism in both. The empirical results above support H2a in this article.

**Table 7:** Test results of the mediating effect based on entrepreneurs’ innovative spirit.

Variables	Macro level			Micro level		
	(1)	(2)	(3)	(4)	(5)	(6)
	GreenIndex1	Esp1	GreenIndex1	GreenIndex2	Esp4	GreenIndex2
Szfix1	0.0325***	0.2947***	0.0293***			
	(21.57)	(6.63)	(11.67)			
Szfix2				0.0405***	1.1393***	0.0363***
				(7.31)	(37.21)	(6.39)
Esp1			0.0070**			
			(2.21)			
Esp4						0.0037***
						(3.32)
Controls	Yes	Yes	Yes	Yes	Yes	Yes
Province/Firm fixed effect	Yes	Yes	Yes	Yes	Yes	Yes
Year fixed effect	Yes	Yes	Yes	Yes	Yes	Yes
R^2^	0.7772	0.6325	0.5080	0.3151	0.0895	0.3152
N	330	330	330	26700	26700	26700
Sobel test	2.078**			3.3499***		
	Effective mechanism -			Effective mechanism -		
	forward conduction mechanism			forward conduction mechanism		

Notes. ***p < 0.01; **p < 0.05; *p < 0.1. The t value is in brackets.

### The intermediary effect based on entrepreneurs’ entrepreneurial spirit

With the development of digitalization, market vitality and consumer demand are further stimulated, resulting in a more abundant number of entrepreneurial opportunities and resources for regions and enterprises. Benefiting from the assistance of digital technology sharing and other characteristics, entrepreneurial entities’ financing constraints and information asymmetry are alleviated. When the entrepreneurial spirit continues to gather, it also forces entrepreneurs to pay attention to the concept of “green” sustainable development, including the environment and society, to establish green development strategies in advance, and to continuously promote the level of green development. According to the empirical results in Table 8, an improvement in the digitalization level at the macro and micro levels can significantly stimulate entrepreneurial spirit, thereby driving the continuous improvement in regional and enterprise green development levels. After Sobel testing, there is a forward conduction mechanism in both cases, and the empirical results above support H2b in this article.

**Table 8:** Test results of the mediating effect based on entrepreneurs’ entrepreneurial spirit.

Variables	Macro level			Micro level		
	(1)	(2)	(3)	(4)	(5)	(6)
	GreenIndex1	Esp2	GreenIndex1	GreenIndex2	Esp5	GreenIndex2
Szfix1	0.0325***	0.0906***	0.0296***			
	(21.57)	(12.69)	(15.95)			
Szfix2				0.0405***	0.3886***	0.0394***
				(7.31)	(5.33)	(7.11)
Esp2			0.0321***			
			(2.64)			
Esp5						0.0028***
						(6.03)
Controls	Yes	Yes	Yes	Yes	Yes	Yes
Province/Firm fixed effect	Yes	Yes	Yes	Yes	Yes	Yes
Year fixed effect	Yes	Yes	Yes	Yes	Yes	Yes
R^2^	0.7772	0.7623	0.7824	0.3151	0.3554	0.1361
N	330	330	330	26700	26700	26700
Sobel test	2.5770***			3.8610***		
	Effective mechanism –			Effective mechanism –		
	forward conduction mechanism			forward conduction mechanism		

Notes.

***p < 0.01; **p < 0.05; *p < 0.1. The t value is in brackets.

### The intermediary effect based on entrepreneurs’ contract spirit

In the era of the digital economy, contract spirit has become an extremely important characteristic in social networks. The formation of contract spirit within a region can reduce the uncertainty and unsafe factors in the process of contract execution. At the same time, it can play a positive role in promoting an improvement in the regional innovation level and achievement transformation, helping the industry create a harmonious development space, ecological environment, and social foundation. For enterprises, digital technologies such as blockchain can be used to quickly establish their own credit database, and entrepreneurs will also pay more attention to their products and commercial civilization images such as "green energy" and "environmental protection and low carbon". According to the empirical analysis results in Table 9, an improvement in the digitalization level at both the macro and micro levels can significantly stimulate entrepreneurs’ contract spirit, thereby driving the continuous improvement in regional and enterprise green development. After Sobel testing, there is a forward conduction mechanism in both cases, and the empirical results above support H2c in this article.

**Table 9:** Test results of the mediating effect based on entrepreneurs’ contract spirit.

Variables	Macro level			Micro level		
	(1)	(2)	(3)	(4)	(5)	(6)
	GreenIndex1	Esp3	GreenIndex1	GreenIndex2	Esp6	GreenIndex2
Szfix1	0.0325***	1.4972***	0.0239***			
	(21.57)	(9.61)	(15.97)			
Szfix2				0.0405***	0.1146***	0.0291***
				(7.31)	(40.91)	(5.10)
Esp3			0.0055***			
			(7.16)			
Esp6						0.0994***
						(8.22)
Controls	Yes	Yes	Yes	Yes	Yes	Yes
Province/Firm fixed effect	Yes	Yes	Yes	Yes	Yes	Yes
Year fixed effect	Yes	Yes	Yes	Yes	Yes	Yes
R^2^	0.7772	0.5173	0.5690	0.3151	0.7560	0.1371
N	330	330	330	26700	26700	26700
Sobel test	5.5914***			8.054***		
	Effective mechanism -			Effective mechanism -		
	forward conduction mechanism			forward conduction mechanism		

Notes.

***p < 0.01; **p < 0.05; *p < 0.1. The T value is in brackets.

## Further analysis

Entrepreneurship affects the development strategies of regions and enterprises. When a subject faces pressure from environmental regulation, the innovative spirit, entrepreneurial spirit, and contract spirit of entrepreneurs will also be guided and constrained in the process of promoting green development. Therefore, this article mainly analyzes the moderating role of environmental regulations. The empirical analysis results are shown in Table 10. For micro enterprises, environmental regulations can positively moderate the promoting effect of entrepreneurs’ innovative spirit, entrepreneurial spirit and contract spirit on green development, and the statistical results are significant at the 1% level. For macro regions, environmental regulations positively moderate the promoting effect of regional entrepreneurs’ contract spirit on green development, but its positive promoting effect on regional entrepreneurs’ innovative spirit and entrepreneurial spirit is not supported by the statistical data in this study. One possible reason is that in the short term, when the innovative and entrepreneurial spirits of regional entrepreneurs gather, the motivation to seize the "fleeting opportunity" increases, and strong environmental regulations increase the transaction costs of innovation entities in the region, causing resistance to the internalization process of negative externalities of environmental pollution in the region. At the same time, there is also a certain lag in the new formats of the energy market and the opportunity space of the green consumption market; thus, if the entire region wants to achieve the "innovation compensation" effect referred to in the "Porter hypothesis" through environmental regulations, it will not be effective in the short term. Therefore, Hypothesis H3 in this article is partially confirmed.

**Table 10:** Test results of the moderating effect of environmental regulations.

Variables	Macro level			Micro level		
	(1)	(2)	(3)	(4)	(5)	(6)
	GreenIndex1	GreenIndex1	GreenIndex1	GreenIndex2	GreenIndex2	GreenIndex2
ER	-5.572***	-0.5345	-0.7706	12.8690***	10.875**	12.4647***
	(-3.161)	(-0.53)	(-0.74)	(5.2)	(2.43)	(2.98)
Esp1	0.012***					
	(-2.996)					
Esp1×ER	-6.098***					
	(-4.603)					
Esp2		0.1578***				
		(7.43)				
Esp2×ER		-16.6045				
		(-0.41)				
Esp3			0.0036***			
			(3.43)			
Esp3×ER			0.3652***			
			(3.11)			
Esp4				0.0015***		
				(4.53)		
Esp4×ER				1.4935***		
				(5.2)		
Esp5					0.0032***	
					(6.77)	
Esp5×ER					0.0752***	
					(3.31)	
Esp6						0.1194***
						(10.12)
Esp6×ER						12.7311***
						(3.98)
Controls	Yes	Yes	Yes	Yes	Yes	Yes
R^2^	0.3450	0.3719	0.2307	0.1355	0.1353	0.1376
N	330	330	330	26608	26608	26608

Notes. ***p < 0.01; **p < 0.05; *p < 0.1. The t value is in brackets.

## Conclusion and suggestions

### Research conclusion

How to promote green and low-carbon development has always been a key issue of concern for scholars and government decision-makers in various countries. This paper explains the intrinsic mechanism of digitalization level affecting the green development from the theoretical level. Then based on the measurement of the digitalization level and green development level of 30 provinces and the listed companies in China, this paper empirically tests the impact of digitalization level on green development and its channels from both the macro and micro levels.

The above research shows the following: (1) The digitalization level has a significant positive promoting effect on the green development of regions and enterprises, and the robustness test verifies the reliability of this conclusion. At the same time, it was found that the blockchain technology has the strongest promoting effect on the green development level of enterprises. (2) Entrepreneurship is the mediating bridge for digitalization level to promote green development. The improvement of the digitalization level promotes the gathering of entrepreneurs’ innovative spirit, entrepreneurial spirit and contract spirit, further promoting the green development of regions and enterprises. (3) For micro enterprises, environmental regulations serve as an "accelerator" of the promoting effect of entrepreneurship on their green development, while environmental regulations plays a significant positive regulatory role in the impact of regional entrepreneurs’ contract spirit on green development.

### Countermeasure suggestions

The findings of this article indicate that in order to leverage the positive impact of digitalization on green development, both governments and enterprises need to accelerate digital construction, integrate and innovate their applications in the fields of resources, energy and environment. They should actively cultivate and promote entrepreneurship, thereby releasing the development dividends of digital technology.

At the macro level, the government should first support the construction of digital infrastructure, accelerate the integration and innovative application of digital technology in the field of ecological environment, precisely plan carbon reduction and carbon neutrality, then appropriately reduce the threshold for industrial digital transformation, continuously optimize the external environment such as the layout and operation mode of digital infrastructure, and lay a solid foundation for the continuous improvement of regional digitalization level. Second, the government should attach importance to the leading role of entrepreneurship in regional green development. On the one hand, the government can provide more opportunities for entrepreneurs to carry out innovation and entrepreneurship activities, and on the other hand, it should also establish a good credit system in the digital economy era. Finally, the government should balance regional innovation development and the constraints brought by environmental regulations and guide the sustainable development of urban green industries and new market opportunities for green consumption from a policy perspective.

At the micro level, enterprises should first continuously improve their digital awareness and digital technology application capabilities, attach importance to cultivating talents who can reasonably apply digital technology such as blockchain, and then accelerate the integration of digital technology in scenarios such as precise measurement and long-term prediction of carbon emissions. Correspondingly, enterprises also need to strengthen research and development investment in digital technology to promote collaborative development of digital technology application innovation and technological innovation. Second, enterprises should actively cultivate and promote entrepreneurship, forming a cultural atmosphere for identifying green innovation opportunities and carrying out green technology innovation activities. At the same time, in the era of the digital economy, a new type of digital trust mechanism has been formed between enterprises and society, enterprises should carry forward the entrepreneurs’ contract spirit, fulfill their commitments to stakeholders, adhere to green and low-carbon concepts when participating in external collaboration, and lead enterprises to build a green development business reputation. Finally, enterprises should actively deal with the pressure and opportunities brought about by environmental regulations, seize the information and impetus provided by the "innovation compensation effect" for technological innovation, and achieve sustainable driving force for green development by building competitive advantages.

## Supporting information

S1 Data (ZIP)
